# Synthesis of *N*-substituted 3-(2-aryl-2-oxoeth­yl)-3-hy­droxy­indolin-2-ones and their conversion to *N*-substituted (*E*)-3-(2-aryl-2-oxo­ethyl­idene)indolin-2-ones: synthetic sequence, spectroscopic characterization and structures of four 3-hy­droxy com­pounds and five oxo­ethyl­idene products

**DOI:** 10.1107/S2053229620004143

**Published:** 2020-04-20

**Authors:** Diana Becerra, Juan Castillo, Braulio Insuasty, Justo Cobo, Christopher Glidewell

**Affiliations:** a Escuela de Ciencias Química, Universidad Pedagógica y Tecnológica de Colombia, 150003 Tunja, Colombia; b Bioorganic Compounds Research Group, Department of Chemistry, Universidad de los Andes, 111711 Bogotá, Colombia; c Heterocyclic Compounds Research Group, Department of Chemistry, Universidad del Valle, AA 25360 Cali, Colombia; d Departamento de Química Inorgánica y Orgánica, Universidad de Jaén, 23071 Jaén, Spain; e School of Chemistry, University of St Andrews, Fife KY16 9ST, Scotland

**Keywords:** synthesis, heterocyclic com­pounds, isatin, 3-hydroxyindolinone, mol­ecular structure, hydrogen bonding, supra­molecular assembly, crystal structure

## Abstract

An operationally simple and time-efficient approach has been developed for the synthesis of racemic *N*-substituted 3-(2-aryl-2-oxoeth­yl)-3-hy­droxy­indolin-2-ones by a piperidine-catalysed aldol reaction between aryl methyl ketones and *N*-alkyl­isatins. These aldol products were used successfully as strategic inter­mediates for the preparation of *N*-substituted (*E*)-3-(2-hetaryl-2-oxo­ethyl­idene)indolin-2-ones by a stereoselective dehydration reaction under acidic conditions. The products have all been fully characterized by ^1^H and ^13^C NMR spectroscopy, by mass spectrometry and, for a representative selection, by crystal structure analysis. In each of (*RS*)-1-benzyl-3-hy­droxy-3-[2-(4-meth­oxy­phen­yl)-2-oxoeth­yl]indolin-2-one, C_24_H_21_NO_4_, (I*c*), and (*RS*)-1-benzyl-3-{2-[4-(di­methyl­amino)­phen­yl]-2-oxoeth­yl}-3-hy­droxy­indolin-2-one, C_25_H_24_N_2_O_3_, (I*d*), inversion-related pairs of mol­ecules are linked by O—H⋯O hydrogen bonds to form *R* ^2^_2_(10) rings, which are further linked into chains of rings by a combination of C—H⋯O and C—H⋯π(arene) hydrogen bonds in (I*c*) and by C—H⋯π(arene) hydrogen bonds in (I*d*). The mol­ecules of (*RS*)-1-benzyl-3-hy­droxy-3-[2-oxo-2-(pyridin-4-yl)eth­yl]indolin-2-one, C_22_H_18_N_2_O_3_, (I*e*), are linked into a three-dimensional framework structure by a combination of O—H⋯N, C—H⋯O and C—H⋯π(arene) hydrogen bonds. (*RS*)-3-[2-(Benzo[*d*][1,3]dioxol-5-yl)-2-oxoeth­yl]-1-benzyl-3-hy­droxy­indolin-2-one, C_24_H_19_NO_5_, (I*f*), crystallizes with *Z*′ = 2 in the space group *P*  and the mol­ecules are linked into com­plex sheets by a combination of O—H⋯O, C—H⋯O and C—H⋯π(arene) hydro­gen bonds. In each of (*E*)-1-benzyl-3-[2-(4-fluoro­phen­yl)-2-oxo­ethyl­idene]indolin-2-one, C_23_H_16_FNO_2_, (II*a*), and (*E*)-1-benzyl-3-[2-oxo-2-(thiophen-2-yl)ethylidene]indolin-2-one, C_21_H_15_NO_2_S, (II*g*), the mol­ecules are linked into simple chains by a single C—H⋯O hydrogen bond, while those of (*E*)-1-benzyl-3-[2-oxo-2-(pyridin-4-yl)ethyl­idene]indolin-2-one, C_22_H_16_N_2_O_2_, (II*e*), are linked by three C—H⋯O hydrogen bonds to form sheets which are further linked into a three-dimensional structure by C—H⋯π(arene) hydrogen bonds. There are no hydrogen bonds in the structures of either (*E*)-1-benzyl-3-[2-(4-meth­oxy­phen­yl)-2-oxo­ethyl­idene]indolin-2-one, C_24_H_19_NO_3_, (II*c*), or (*E*)-1-benzyl-5-chloro-3-[2-(4-chloro­phen­yl)-2-oxo­ethyl­idene]indolin-2-one, C_23_H_15_Cl_2_NO_2_, (II*h*), but the mol­ecules of (II*h*) are linked into chains of π-stacked dimers by a combination of C—Cl⋯π(arene) and aromatic π–π stacking inter­actions.

## Abstract 2

Ten novel 3-(2-aryl-2-oxoeth­yl)-3-hy­droxy­indolin-2-ones have been synthesized, mostly in excellent yield, and characterized spectroscopically, and nine of these have been dehydrated to the corresponding (*E*)-3-(2-aryl-2-oxo­ethyl­idene)indolin-2-ones, also in excellent yield, and again characterized spectroscopically. The structures of four of the former and five of the latter are reported, and all show different patterns of supra­molecular assembly.

## Introduction   

Almost 60% of drugs based on small organic mol­ecules which are in use for medicinal purposes contain at least one N-heterocyclic ring (Vitaku *et al.*, 2014 ▸). Amongst these, isatin (1*H*-indole-2,3-dione) and its derivatives have attracted 
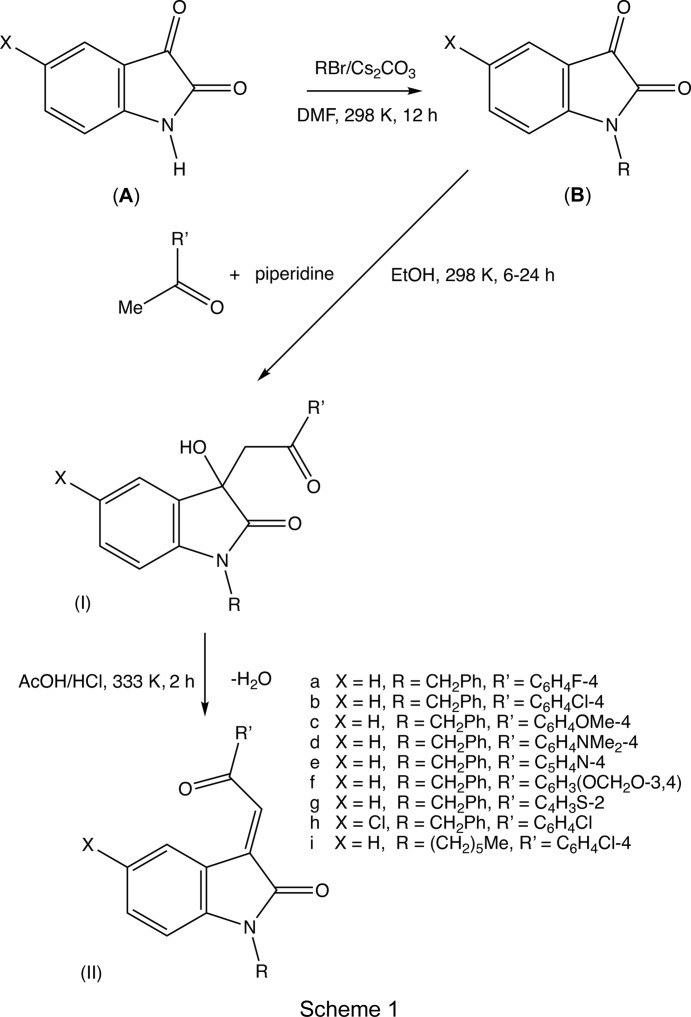
particular inter­est because of their broad range of biological and pharmacological activities (Singh & Desta, 2012 ▸; Pakravan *et al.*, 2013 ▸). Isatin derivatives have also been found to be useful synthetic inter­mediates for the production of both dyestuffs and organic electronic materials (Stalder *et al.*, 2014 ▸; Deng & Zhang, 2014 ▸). These wide-ranging applications have prompted the development of a large range of synthetic approaches to functionalized isatin derivatives (Moradi *et al.*, 2017 ▸; Bogdanov & Mironov, 2018 ▸; Varun *et al.*, 2019 ▸). Amongst these, the addition of nucleophilic units to the pro­chiral carbonyl group at atom C3 permits the construction of chiral 3-substituted-3-hy­droxy­indolin-2-ones containing a stereo­genic centre at the 3-position (Peddibhotla, 2009 ▸; Mohammadi *et al.*, 2013 ▸). Such species are desirable targets, because many related structural motifs are found in natural products and pharmaceutically active com­pounds; for example, convolutamydine A is a bioactive alkaloid with significant activity against HL-60 human plomyelocytic leukemia cells (Kamano *et al.*, 1995 ▸), SM-130686 is a novel orally active growth hormone secretagogue (Nagamine *et al.*, 2001 ▸), donaxaridine has shown effective anti­cancer properties (Kimura *et al.*, 2016 ▸) and maremycins A and B exhibit anti­bacterial, anti­fungal and anti­tumour properties (Duan *et al.*, 2018 ▸).

Several years ago, we reported the synthesis and structures of a range of 3-alkyl-3-hy­droxy­indolin-2-ones by reaction of isatin itself with a variety of methyl ketones in the presence of piperidine. Although the procedures were straightforward, the yields were consistently rather disappointing, in the range 40–60% (Becerra *et al.*, 2010 ▸). Within the isatin mol­ecule, both simple amide and vinyl­ogous amide fragments can be identified, so that in the conjugate base of isatin the negative charge can be delocalized into both carbonyl groups. Any consequent partial proton transfer from isatin to piperidine is thus likely to be a factor in depressing the overall yields. We therefore reasoned that incorporation of a substituent at the N atom of isatin should prevent any such ionization and thus increase the product yields significantly.

Accordingly, we have now studied the synthesis and structures of a range of 3-(2-aryl-2-oxoeth­yl)-3-hy­droxy­indolin-2-ones, (I), and their dehydration to the corresponding chalcones, (II) (see Scheme 1), and we report here the synthesis and spectroscopic characterization of nine com­pounds of type (I), and eight of type (II), along with the mol­ecular and supra­molecular structures of four representative type (I) com­pounds [(I*c*), (I*d*), (I*e*) and (I*f*)] and of five representative type (II) com­pounds [(II*a*), (II*c*), (II*e*), (II*g*) and (II*h*)].

## Experimental   

### Synthesis and crystallization   

Isatins (**A**) (see Scheme 1), where *X* = H or Cl, were converted to the corresponding *N*-alkyl analogues (**B**) by reaction with the appropriate alkyl bromide in di­methyl­formamide solution in the presence of solid caesium carbonate acting as a weak base, giving yields in excess of 90% after a reaction time of 12 h at 298 K. For the synthesis of the 3-hy­droxy­indolin-2-ones, (I) (see Scheme 1), a mixture of an *N*-alkyl­isatin, (**B**) (1.0 mmol), the appropriate aryl methyl ketone (1.0 mmol) and piperidine (0.2 mmol) in ethanol (10 ml) was stirred at 298 K for 6 h [24 h in the case of com­pound (I*d*)], after which time the starting materials were no longer detectable using thin-layer chromatography (TLC). The resulting solid products were collected by filtration, washed with cold ethanol (2 ml) and dried in air to give the products of type (I). Analytical data for com­pound (I*a*): yield 93%, m.p. 437–438 K (literature 437–441 K; Tripathi *et al.*, 2016 ▸); com­pound (I*b*): yield 91%, m.p. 430–431 K (literature 431–433 K; Duan *et al.*, 2013 ▸); com­pound (I*c*): yield 85%, m.p. 430 K (literature 429–431 K; Duan *et al.*, 2013 ▸); com­pound (I*d*): yield 36%, m.p. 450 K; com­pound (I*e*): yield 92%, m.p. 479–481 K; com­pound (I*f*): yield 87%, m.p. 452–453 K; com­pound (I*g*): yield 82%, m.p. 421–422 K (literature 421–423 K; Satish *et al.*, 2015 ▸); com­pound (I*h*): yield 89%, m.p. 423 K; com­pound (I*i*): yield 83%, m.p. 398 K. Colourless crystals of com­pounds (I*c*), (I*d*), (I*e*) and (I*f*) suitable for single-crystal X-ray diffraction analysis were grown by slow evaporation, at ambient temperature and in the presence of air, of solutions in ethanol–di­methyl­formamide (6:1 *v*/*v*).

For the conversion of the 3-hy­droxy com­pounds (I) into the ethyl­idene products (II), a solution of the appropriate 3-hy­droxy com­pound (I) (0.50 mmol) in glacial acetic acid (2 ml) was stirred at ambient temperature for 10 min, during which time concentrated hydro­chloric acid (0.1 ml) was added slowly. The resulting mixtures were then stirred at 333 K for a further 2 h. For each mixture, the pH was then brought to 7.0 by the addition of a concentrated aqueous solution of ammonia, and the resulting solid products were collected by filtration, washed with cold water and dried in air to give the products of type (II). Analytical data for com­pound (II*a*): yield 92%, m.p. 425–426 K; com­pound (II*b*): yield 85%, m.p. 417–418 K; com­pound (II*c*): yield 93%, m.p. 393 K; com­pound (II*e*): yield 97%, m.p. 398 K; com­pound (II*f*): yield 88%, m.p. 388 K; com­pound (II*g*): yield 91%, m.p. 388–389 K; com­pound (II*h*): yield 89%, m.p. 401–403 K; com­pound (II*i*): yield 83%, m.p. 376–378 K. Crystals of com­pounds (II*a*) (orange), and (II*b*), (II*c*), (II*e*) and (II*g*) (all red) suitable for single-crystal X-ray diffraction analysis were grown by slow evaporation, at ambient temperature and in the presence of air, of solutions in ethanol–di­methyl­formamide (initial com­position 6:1 *v*/*v*).

Spectroscopic data for com­pounds (I*a*), (I*b*), (I*c*) and (I*g*) have been reported recently in the literature (Duan *et al.*, 2013 ▸; Satish *et al.*, 2015 ▸; Tripathi *et al.*, 2016 ▸). Spectroscopic characterization data (^1^H and ^13^C NMR, and mass spectra) for the other com­pounds reported here are provided in the supporting information.

### Refinement   

Crystal data, data collection and structure refinement details are summarized in Table 1 ▸. Compound (II*a*) was handled as a non-merohedral twin with the twin matrix (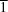
00 0
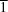
0 0.350,0,1) and with refined twin fractions of 0.1953 (14) and 0.8047 (14). Compound (II*e*) was also handled as a non-merohedral twin, with the twin matrix (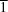
00 0
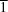
0 0.989,0,1) and refined twin fractions of 0.9654 (6) and 0.0346 (6). In com­pound (II*g*), the thio­phene unit is disordered over two sets of atomic sites having unequal occupancies for the minor-disorder com­ponent, and the bonded and [1,2]-nonbonded distances were restrained to be the same as the corresponding distances in the major-disorder com­ponent, subject to s.u. values of 0.01 and 0.02 Å, respectively; in addition, the anisotropic displacement parameters of pairs of partial-occupancy atoms occupying essentially the same physical space were constrained to be equal. All H atoms, apart from those in the minor-disorder com­ponent of com­pound (II*g*), were located in difference maps. H atoms bonded to C atoms were then treated as riding atoms in geometrically idealized positions, with C—H = 0.95 (alkenyl, aryl and heteroar­yl), 0.98 (CH_3_) or 0.99 Å (CH_2_) and with *U*_iso_(H) = *kU*_eq_(C), where *k* = 1.5 for the methyl groups, which were permitted to rotate but not to tilt, and 1.2 for all other H atoms bonded to C atoms; the H atoms in the minor-disorder com­ponent of com­pound (II*g*) were included on the same basis, giving refined disorder occupancies of 0.9387 (19) and 0.0613 (19). For the H atoms bonded to O atoms, the atomic coordinates were refined with *U*_iso_(H) = 1.5*U*_eq_(O), giving the O—H distances shown in Table 2 ▸.

## Results and discussion   

**Figure 1:**
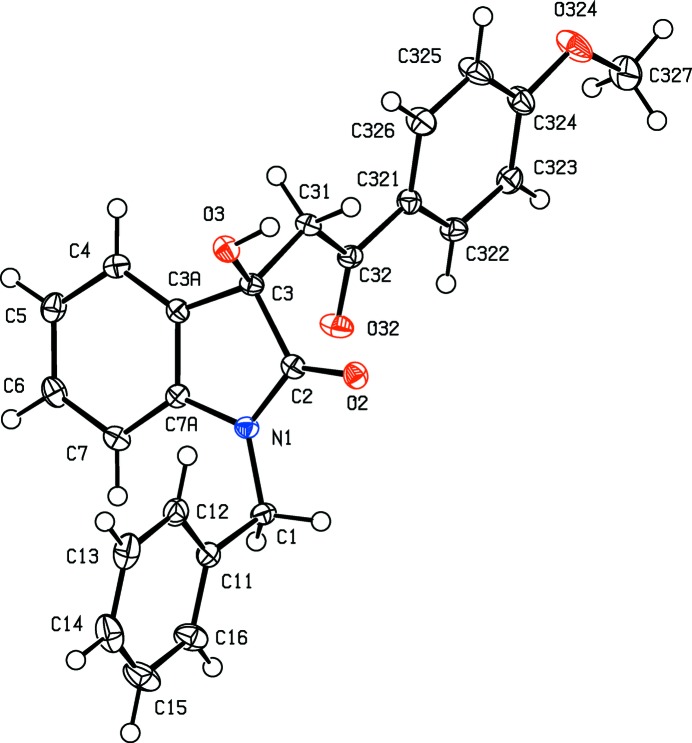
The mol­ecular structure of the *R* enanti­omer of com­pound (I*c*), showing the atom-labelling scheme. Displacement ellipsoids are drawn at the 30% probability level.

**Figure 2:**
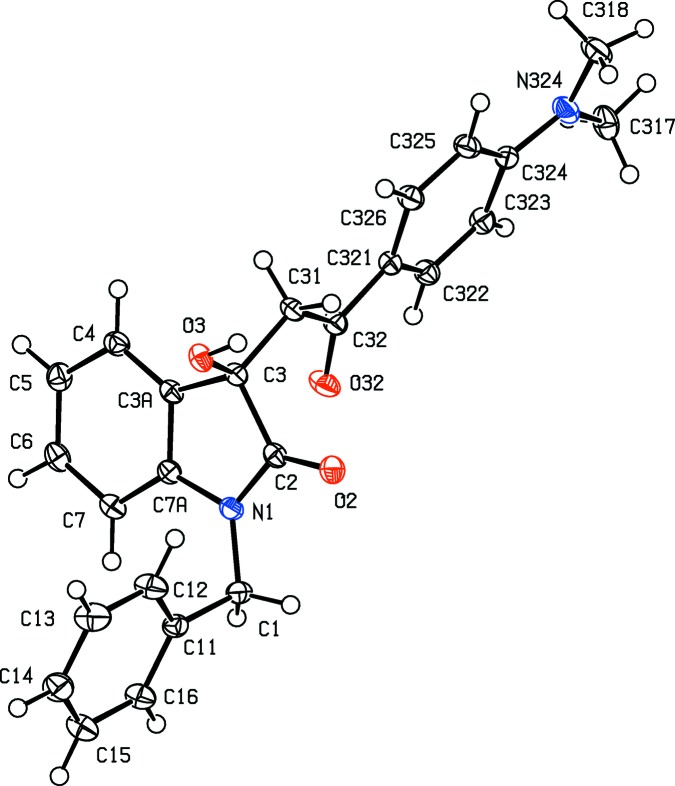
The mol­ecular structure of the *R* enanti­omer of com­pound (I*d*), showing the atom-labelling scheme. Displacement ellipsoids are drawn at the 30% probability level.

**Figure 3:**
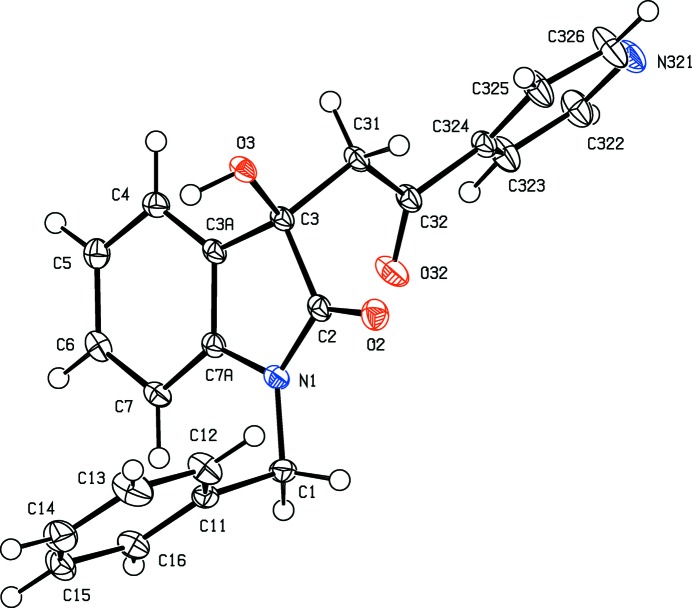
The mol­ecular structure of the *R* enanti­omer of com­pound (I*e*), showing the atom-labelling scheme. Displacement ellipsoids are drawn at the 30% probability level.

**Figure 4:**
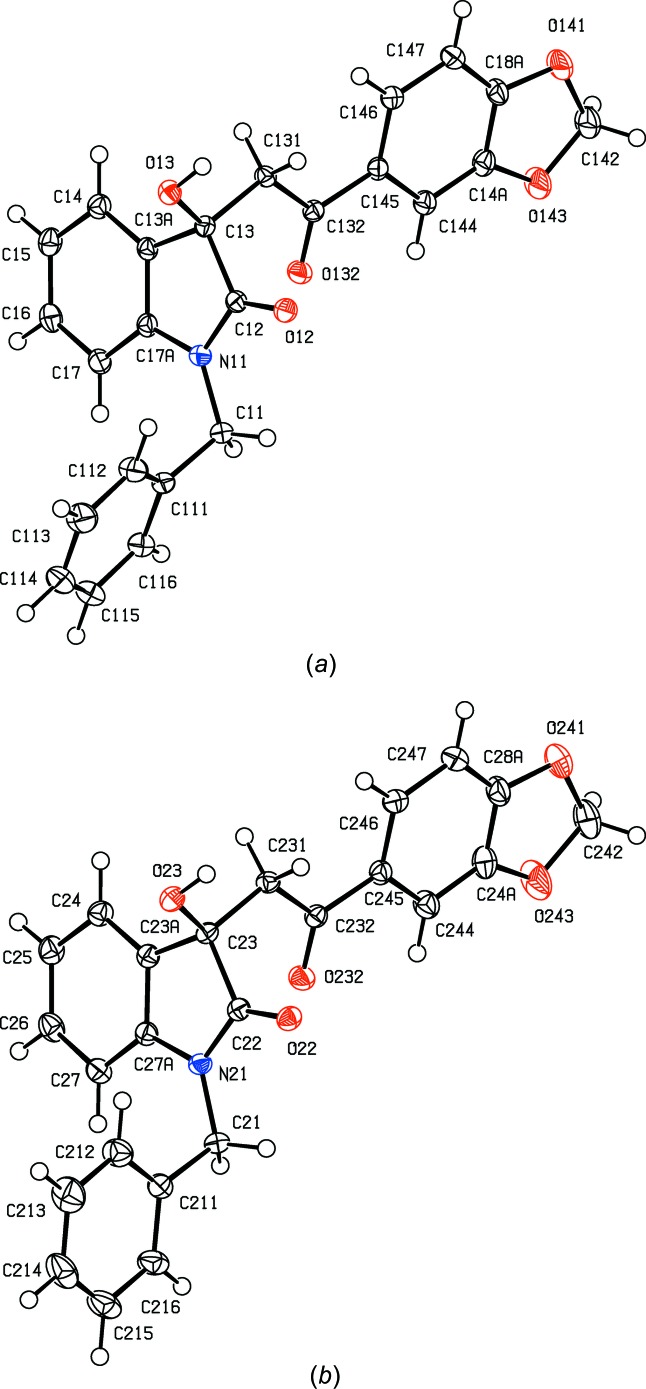
The *R* enanti­omers of the two independent mol­ecules of com­pound (I*f*), showing the atom-labelling schemes for (*a*) mol­ecule 1 and (*b*) mol­ecule 2. Displacement ellipsoids are drawn at the 30% probability level.

**Figure 5:**
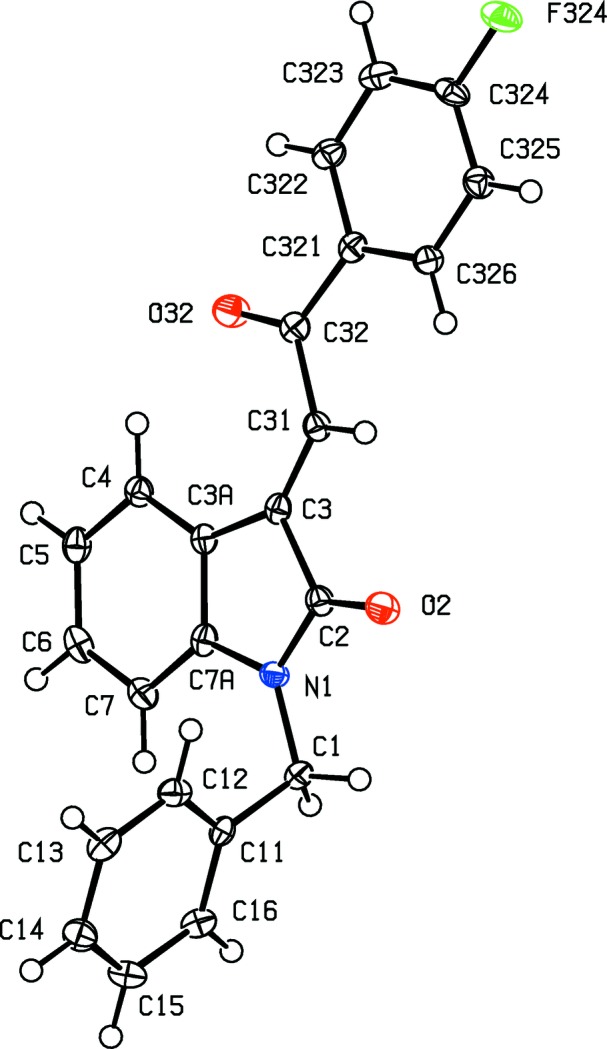
The mol­ecular structure of com­pound (II*a*), showing the atom-labelling scheme. Displacement ellipsoids are drawn at the 30% probability level.

**Figure 6:**
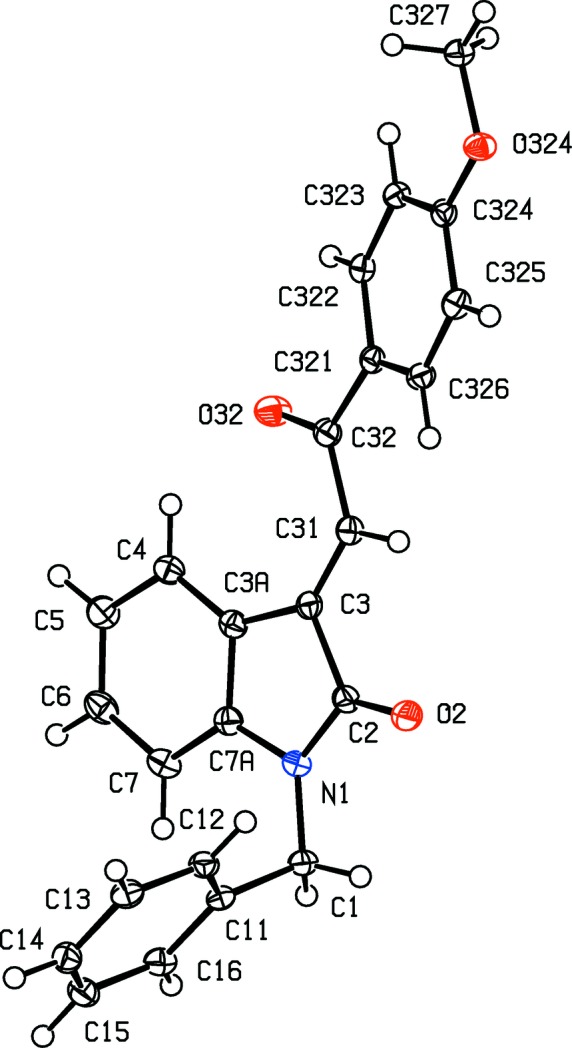
The mol­ecular structure of com­pound (II*c*), showing the atom-labelling scheme. Displacement ellipsoids are drawn at the 30% probability level.

**Figure 7:**
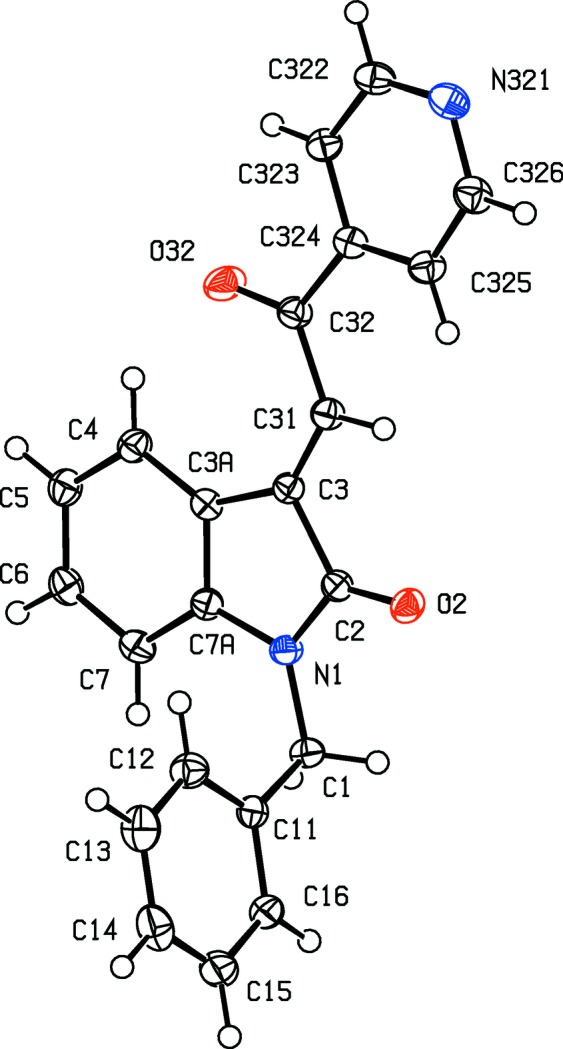
The mol­ecular structure of com­pound (II*e*), showing the atom-labelling scheme. Displacement ellipsoids are drawn at the 30% probability level.

**Figure 8:**
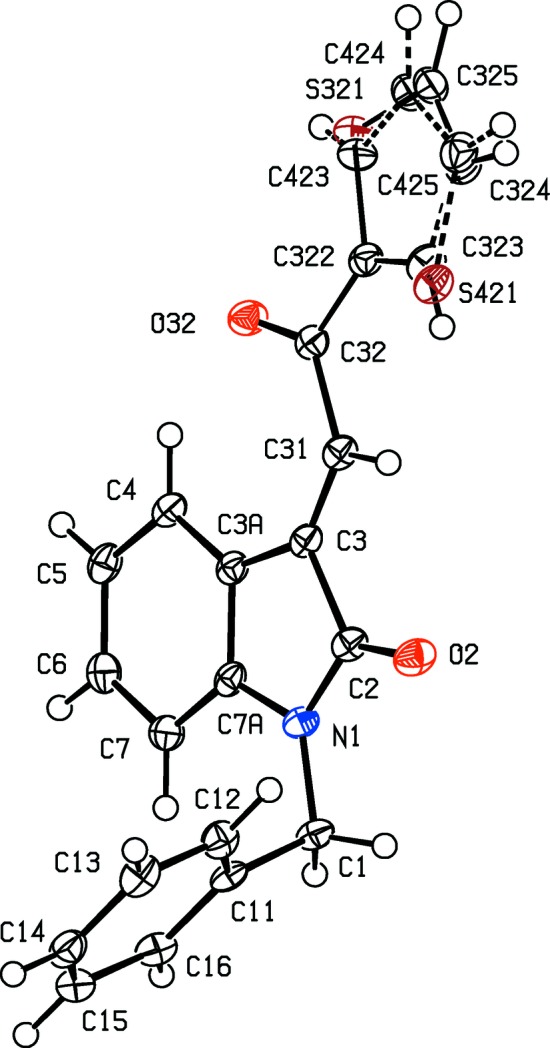
The mol­ecular structure of com­pound (II*g*), showing the atom-labelling scheme and the disorder of the thio­phene unit. The major-disorder com­ponent is drawn using full lines and the minor-disorder com­ponent has been drawn using broken lines. Displacement ellipsoids are drawn at the 30% probability level.

**Figure 9:**
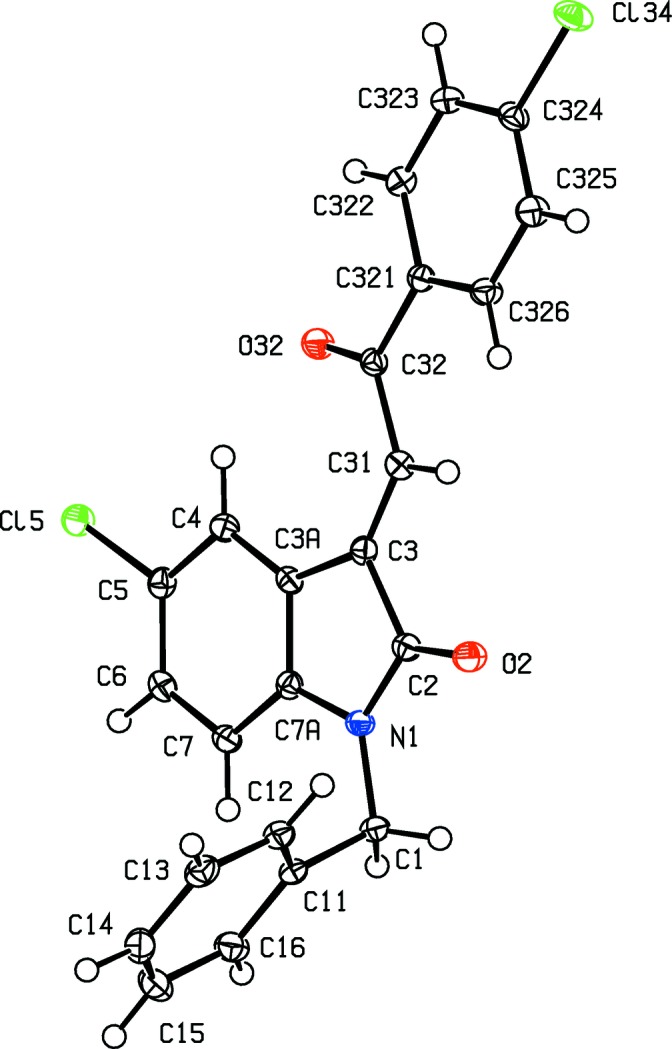
The mol­ecular structure of com­pound (II*h*), showing the atom-labelling scheme. Displacement ellipsoids are drawn at the 30% probability level.

The title com­pounds were synthesized starting from the readily available isatins (**A**), (see Scheme 1, where *X* = H or Cl; Figs. 1–9 ▸ ▸ ▸ ▸ ▸ ▸ ▸ ▸ ▸). The *N*-alkyl­ation of the starting isatins was explored using both benzyl bromide and 1-hexyl bromide in the presence of caesium carbonate as a weak non-nucleophilic base, giving isolated yields of the *N*-alkyl inter­mediates (**B**) consistently in excess of 90%. Focusing primarily on the N-benzyl inter­mediate of type (**B**), the subsequent reactions with aryl methyl ketones in the presence of piperidine did indeed provide generally much higher yields of the products of type (I), usually well above 80%, than had previously been achieved using isatin carrying no substituent at the N atom, which is consistent with the idea of partial proton transfer from the *N*-unsubstituted isatin to piperidine. The yields in both steps appear to be much the same regardless of whether the substituent at the N atom is benzyl or 1-hexyl, or whether the substituent at C5 is H or Cl. The only exception was found for com­pound (I*d*), where the yield was quite low, 36%, even after a much longer reaction time than that required for all the other type (I) products; this may be associated with the strongly electron-donating nature of the di­methyl­amino group. Acid-catalysed dehydration of nine of products (I) gave the *N*-substituted (*E*)-3-(2-aryl-2-oxo­ethyl­idene)indolin-2-ones (II), again with yields well above 80%, although, because of the slow formation and poor yields of (I*d*) in the first step, the dehydration of this inter­mediate was not pursued.

For all of the products of types (I) and (II) (see Scheme 1), the mass spectra confirm their overall com­positions, while the NMR spectra contain all of the expected signals, thus confirming that all the reactions have proceeded as expected and confirming the identity of the products. Each of the aldol products of type (I) contains a stereogenic centre, at atom C3 in (I*c*), (I*d*) and (I*e*), and at atoms C13 and C23 in the two independent mol­ecules in (I*f*). In every case, the reference mol­ecule was selected as one having the *R* configuration at this atom, but the space groups (Table 1 ▸) confirm that, in each case, the com­pound has crystallized as a racemic mixture; in the absence from the synthesis of any agent capable of inducing enanti­oselectivity, it can confidently assumed that all of the other products of type (I) are also formed as racemic mixtures. For each of the type (II) products, only a single geometric isomer was isolated, with no chromatographic or spectroscopic evidence for the formation of even traces of any second isomer. As well as confirming the identity and racemic nature of the type (I) products, the crystal structure analyses have established that in each of the products of type (II) examined here the chalcone unit has the *E* configuration.

In each of the aldol com­pounds of type (I) (Figs. 1 ▸–4 ▸ ▸ ▸), the orientation of the *N*-benzyl unit relative to the indolinone nucleus shows some variation, as indicated by the key torsion angles (Table 3 ▸), despite the fact that atoms from the benzyl unit participate in inter­molecular hydrogen bonding only in aldol (I*c*) (Table 2 ▸). Similar variations in the orientation of the benzyl group are found in the chalcones of type (II) (Table 4 ▸), where this unit participates in inter­molecular hydrogen bonding in both (II*a*) and (II*e*), but not in any of (II*c*), (II*g*) and (II*h*). On the other hand, the conformations of the rest of the mol­ecule relative to the indolinone unit is broadly similar in both type (I) and type (II) com­pounds, aside from the pyridyl derivative (I*e*). In each of 4-meth­oxy derivatives (I*c*) and (II*c*), the two exocyclic C—C—O angles at atom C324 (Figs. 1 ▸ and 6 ▸) differ by *ca* 10°, as typically found (Seip & Seip, 1973 ▸; Ferguson *et al.*, 1996 ▸) for near-planar alk­oxy­arene units; the deviations of methyl atoms C327 from the planes of the adjacent aryl rings are only 0.112 (3) and 0.183 (2) Å in (I*c*) and (II*c*), respectively.

**Figure 10:**
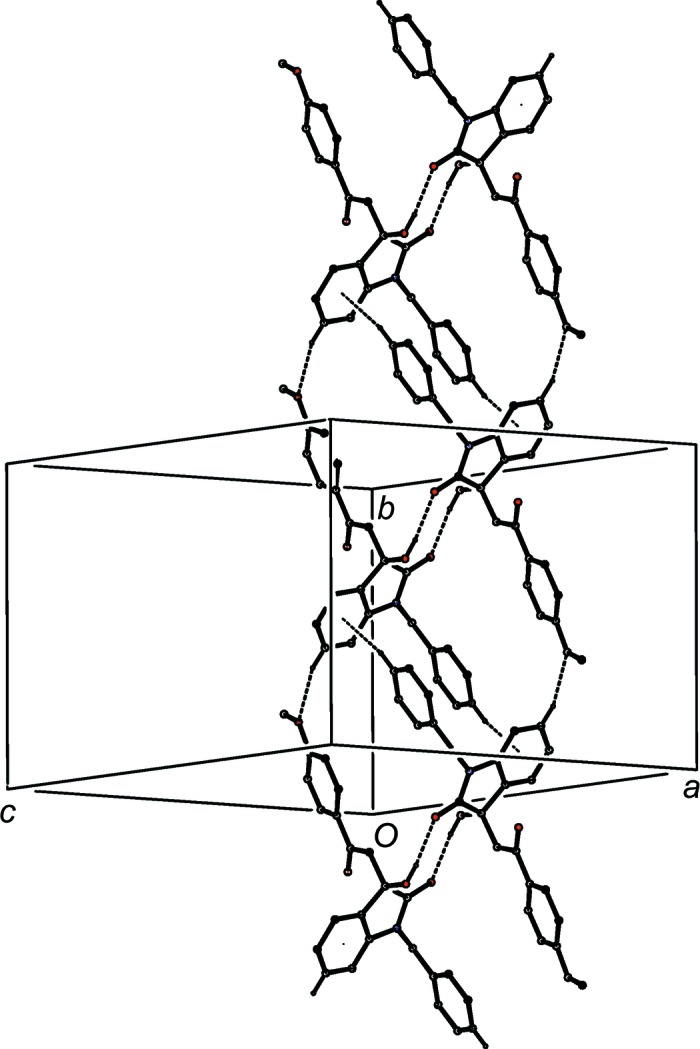
Part of the crystal structure of com­pound (I*c*), showing the formation of a chain of rings built from O—H⋯O, C—H⋯O and C—H⋯π(arene) hydrogen bonds. Hydrogen bonds are drawn as dashed lines and, for the sake of clarity, H atoms not involved in the motifs shown have been omitted.

The mol­ecules of com­pound (I*c*) are linked into a chain of rings by a combination of O—H⋯O, C—H⋯O and C—H⋯π(arene) hydrogen bonds (Table 2 ▸). Inversion-related pairs of mol­ecules are linked by pairs of O—H⋯O hydrogen bonds, forming an 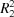
(10) ring (Etter, 1990 ▸; Etter *et al.*, 1990 ▸; Bernstein *et al.*, 1995 ▸) centred at (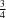, 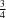, 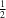). A second centrosymmetric motif, now centred at (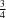, 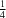, 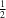), is generated by the C—H⋯π(arene) hydrogen bond having atom C14 as the donor and, in combination, these two motifs generate a chain of rings running parallel to the [010] direction, in which the 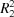
(10) rings centred at (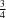, 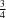 + *n*, 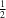) alternate with the rings generated by C—H⋯π hydrogen bonds, centred at (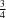, 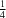 + *n*, 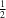), where *n* represents an integer in each case. The chain formation is augmented by a C—H⋯O hydrogen bond having atom C6 as the donor and linking mol­ecules which are related by translation into a *C*(13) chain motif (Fig. 10 ▸). There are two other short inter­molecular contacts in the structure, involving atoms C4 and C31, but these are unlikely to be of real structural significance (Wood *et al.*, 2009 ▸).

**Figure 11:**
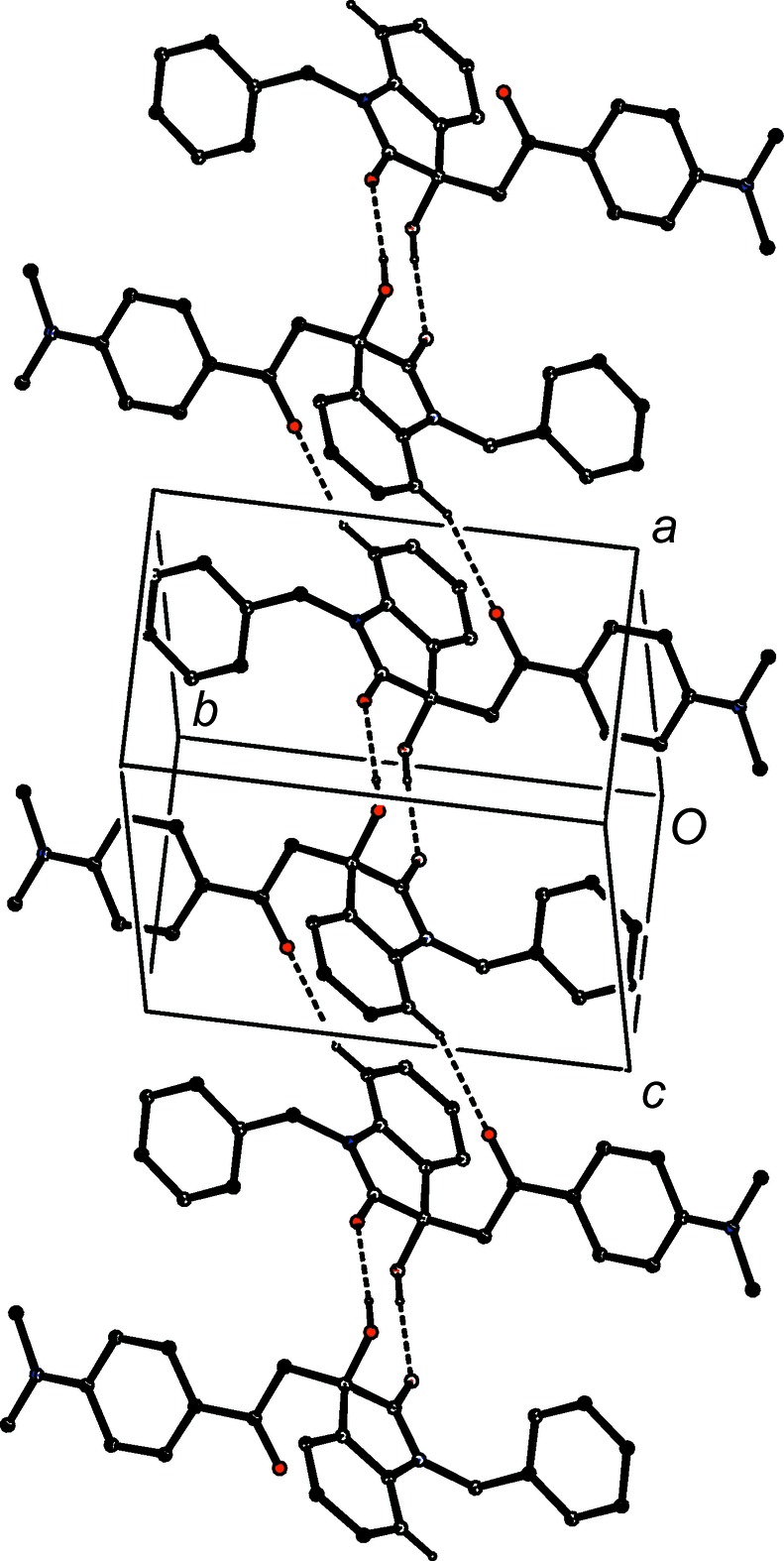
Part of the crystal structure of com­pound (I*d*), showing the formation of a chain of rings built from O—H⋯O and C—H⋯O hydrogen bonds. Hydrogen bonds are drawn as dashed lines and, for the sake of clarity, H atoms not involved in the motifs shown have been omitted.

The supra­molecular assembly in com­pound (I*d*) is very simple, taking the form of a chain of rings running parallel to the [10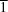] direction (Fig. 11 ▸). Rings of 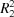
(10) type, containing O—H⋯O hydrogen bonds (Table 2 ▸) and centred at (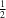 + *n*, 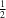, 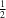 − *n*) alternate with rings of 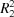
(16) type, containing C—H⋯O hydrogen bonds and centred at (*n*, 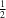, 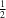 − *n*), where *n* represents an integer in each case. There is a long C—H⋯π(arene) contact within the chain, but there are no direction-specific inter­actions between adjacent chains.

**Figure 12:**
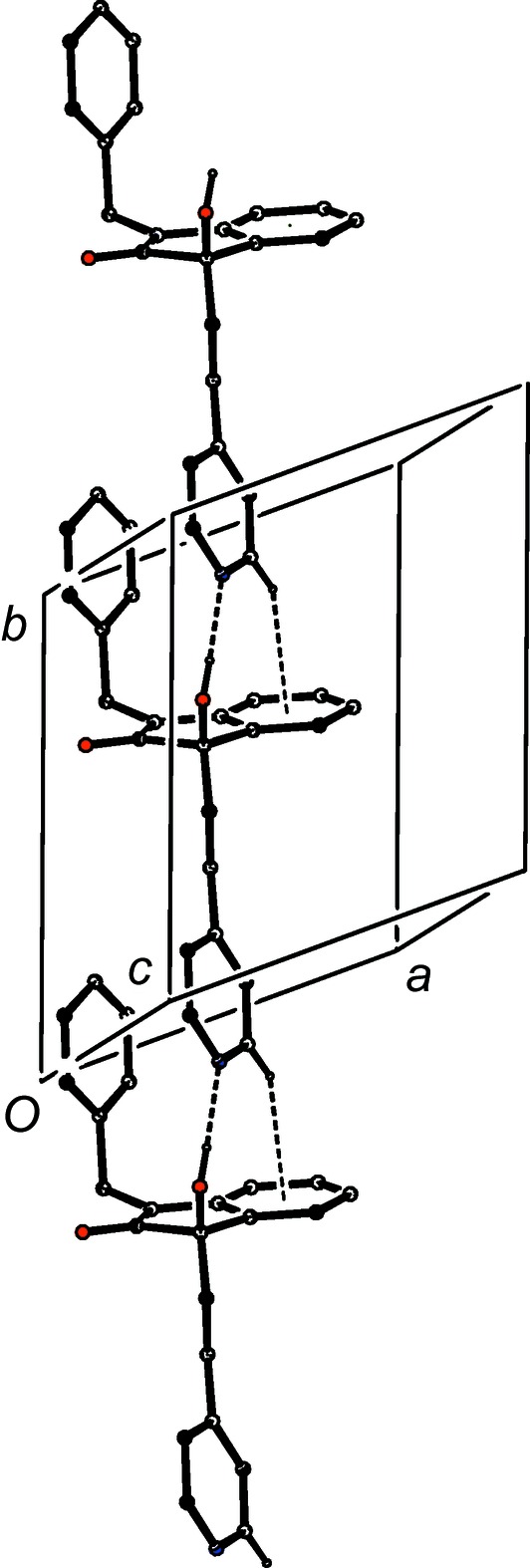
Part of the crystal structure of com­pound (I*e*), showing the formation of a chain of rings running parallel to the [010] direction and built from O—H⋯N and C—H⋯π(arene) hydrogen bonds. Hydrogen bonds are drawn as dashed lines and, for the sake of clarity, H atoms not involved in the motifs shown have been omitted.

**Figure 13:**
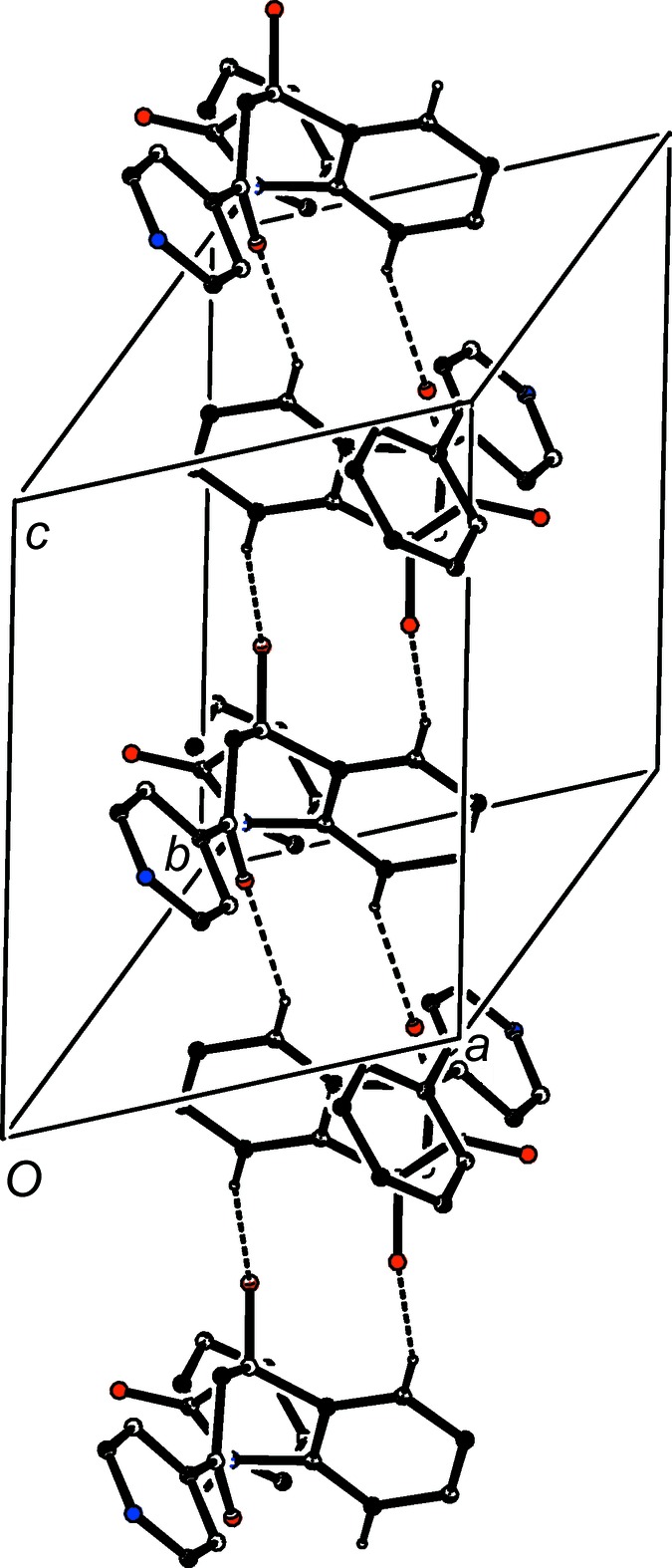
Part of the crystal structure of com­pound (I*e*), showing the formation of a chain of rings running parallel to the [001] direction and built from two types of C—H⋯O hydrogen bonds. Hydrogen bonds are drawn as dashed lines and, for the sake of clarity, H atoms not involved in the motifs shown have been omitted.

**Figure 14:**
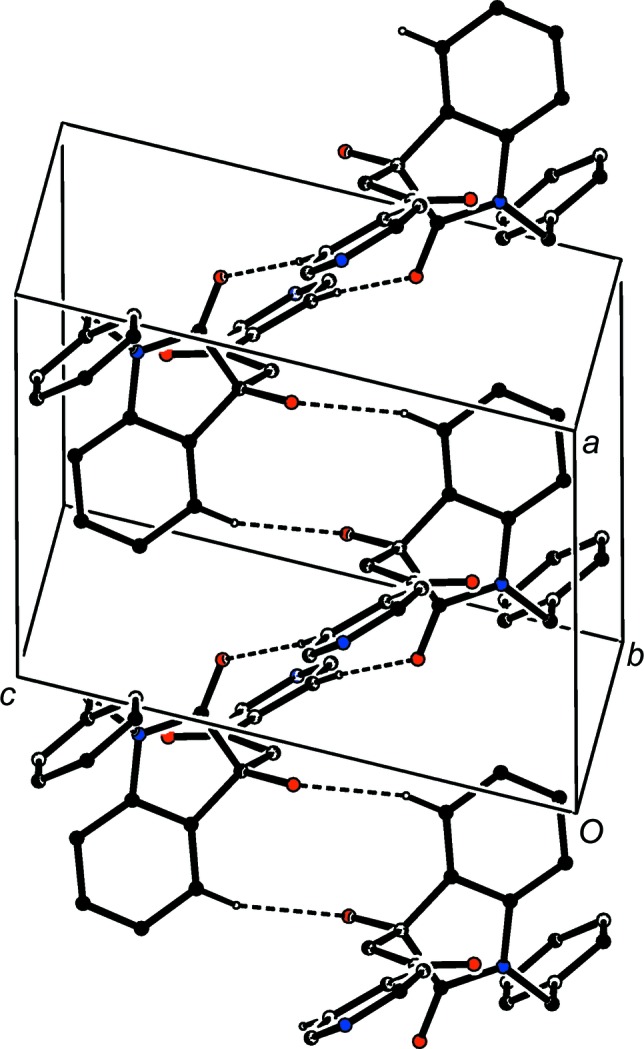
Part of the crystal structure of com­pound (I*e*), showing the formation of a chain of rings running parallel to the [100] direction and built from two types of C—H⋯O hydrogen bonds. Hydrogen bonds are drawn as dashed lines and, for the sake of clarity, H atoms not involved in the motifs shown have been omitted.

In contrast to the simplicity of the assembly in 4-(di­methyl­amino)­phenyl com­pound (I*d*), that in 4-pyridine derivative (I*e*) takes the form of the three-dimensional framework structure built from O—H⋯N, C—H⋯π(arene) and multiple C—H⋯O hydrogen bonds (Table 2 ▸), but the formation of the framework structure is readily analysed in terms of three one-dimensional substructures (Ferguson *et al.*, 1998*a* ▸,*b* ▸; Gregson *et al.*, 2000 ▸). A combination of O—H⋯N and C—H⋯π(arene) hydrogen bonds links mol­ecules which are related by translation into a chain of rings running parallel to the [010] direction (Fig. 12 ▸). In the second substructure, a combination of the two hydrogen bonds having atoms C4 and C7 as the donors generates a chain of centrosymmetric rings running parallel to the [001] direction, in which 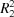
(10) rings centred at (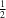, 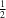, 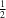 + *n*) alternate with 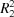
(16) rings centred at (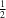, 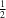, *n*), where *n* represents an integer (Fig. 13 ▸). In the final substructure, the combination of the two hydrogen bonds having atoms C4 and C322 as the donors generates a chain of centrosymmetric rings running parallel to the [100] direction, in which 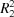
(10) rings centred at (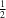 + *n*, 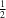, 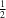) alternate with 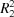
(16) rings centred at (*n*, 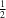, *n*), where *n* represents an integer (Fig. 14 ▸). The combination of chains along [100], [010] and [001] suffices to generate the three-dimensional framework structure.

**Figure 15:**
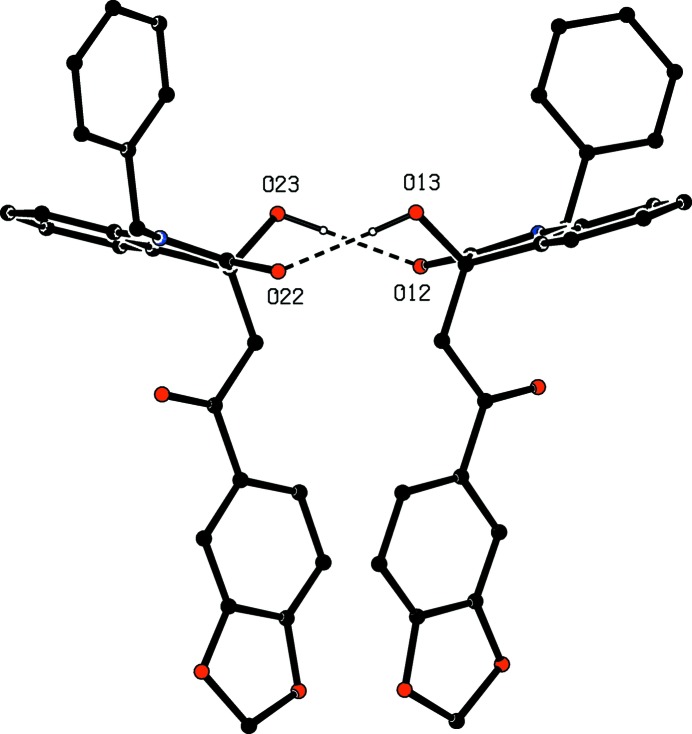
Part of the crystal structure of com­pound (I*f*), showing the linking of the two independent mol­ecules by two independent O—H⋯O hydrogen bonds. Hydrogen bonds are drawn as dashed lines and, for the sake of clarity, H atoms not involved in the motifs shown have been omitted.

**Figure 16:**
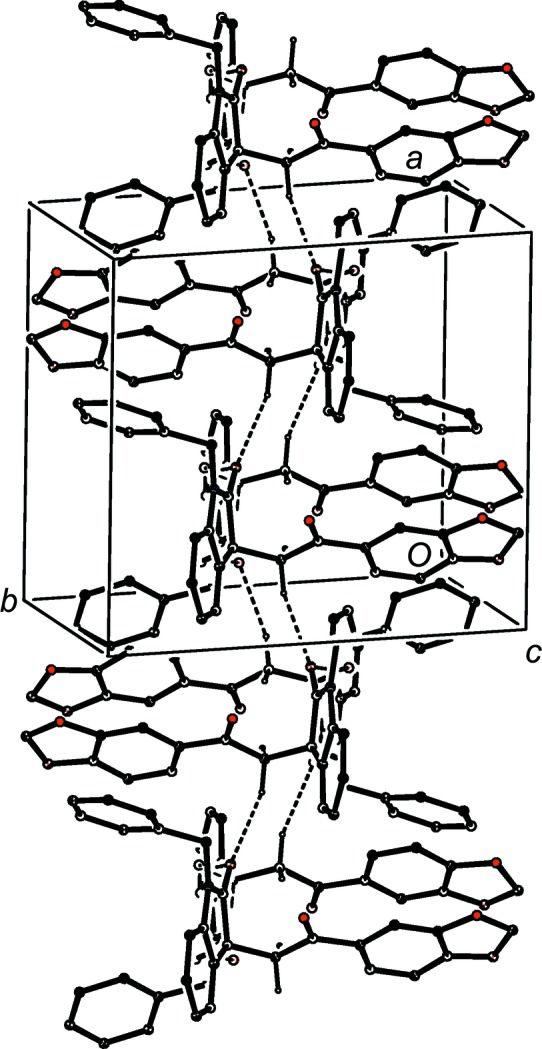
Part of the crystal structure of com­pound (I*f*), showing the formation of a chain of rings running parallel to the [100] direction and built from O—H⋯O and C—H⋯O hydrogen bonds. Hydrogen bonds are drawn as dashed lines and, for the sake of clarity, H atoms not involved in the motifs shown have been omitted.

**Figure 17:**
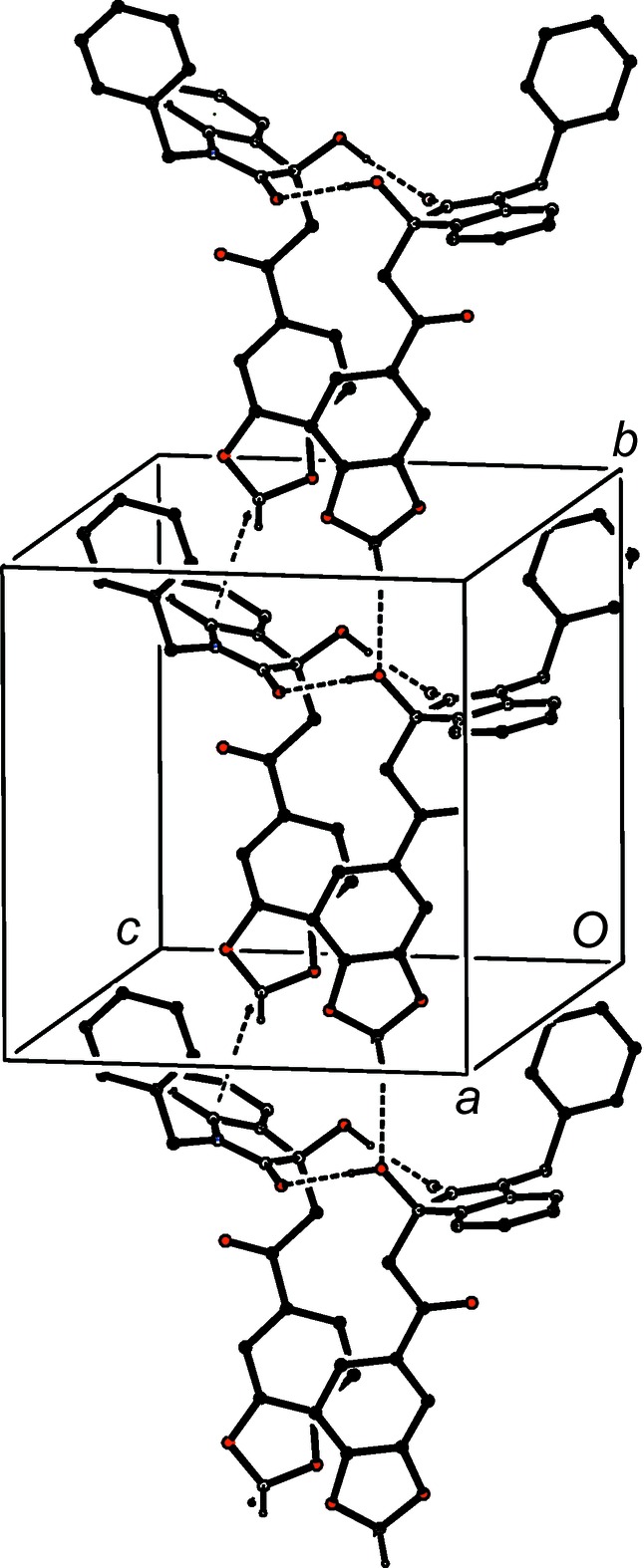
Part of the crystal structure of com­pound (I*f*), showing the formation of a chain of rings running parallel to the [010] direction and built from O—H⋯O, C—H⋯O and C—H⋯π(arene) hydrogen bonds. Hydrogen bonds are drawn as dashed lines and, for the sake of clarity, H atoms not involved in the motifs shown have been omitted.

Compound (I*f*) crystallizes with two mol­ecules in the asymmetric unit but, despite this and the resulting number of independent hydrogen bonds (Table 2 ▸), the supra­molecular assembly in only two-dimensional and, as with (I*e*), this can be analysed in terms of low-dimensional substructures. The two mol­ecules within the selected asymmetric unit (Fig. 15 ▸) are linked by two O—H⋯O hydrogen bonds to form a dimeric unit having approximate, but noncrystallographic, twofold rotation symmetry (Fig. 15 ▸). This finite, or zero-dimensional, substructure can conveniently be regarded as the basic building block in the supra­molecular assembly. These dimeric units are linked by the C—H⋯O hydrogen bonds having atoms C131 and C231 as the donors to form a chain of rings running parallel to the [100] direction, in which 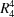
(12) rings centred at (*n*, 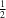, 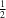) alternate with 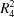
(12) rings centred at (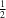 + *n*, 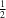, 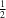), where *n* represents an integer in each case (Fig. 16 ▸). A second one-dimensional substructure arises from the linking of dimeric units which are related by translation by a combination of C—H⋯O and C—H⋯π(arene) hydrogen bonds to form a second chain of rings, this time running parallel to the [010] direction (Fig. 17 ▸). The combination of chains along [100] and [010] gives rise to com­plex sheets lying parallel to (001), but there are not direction-specific inter­actions between adjacent sheets.

**Figure 18:**
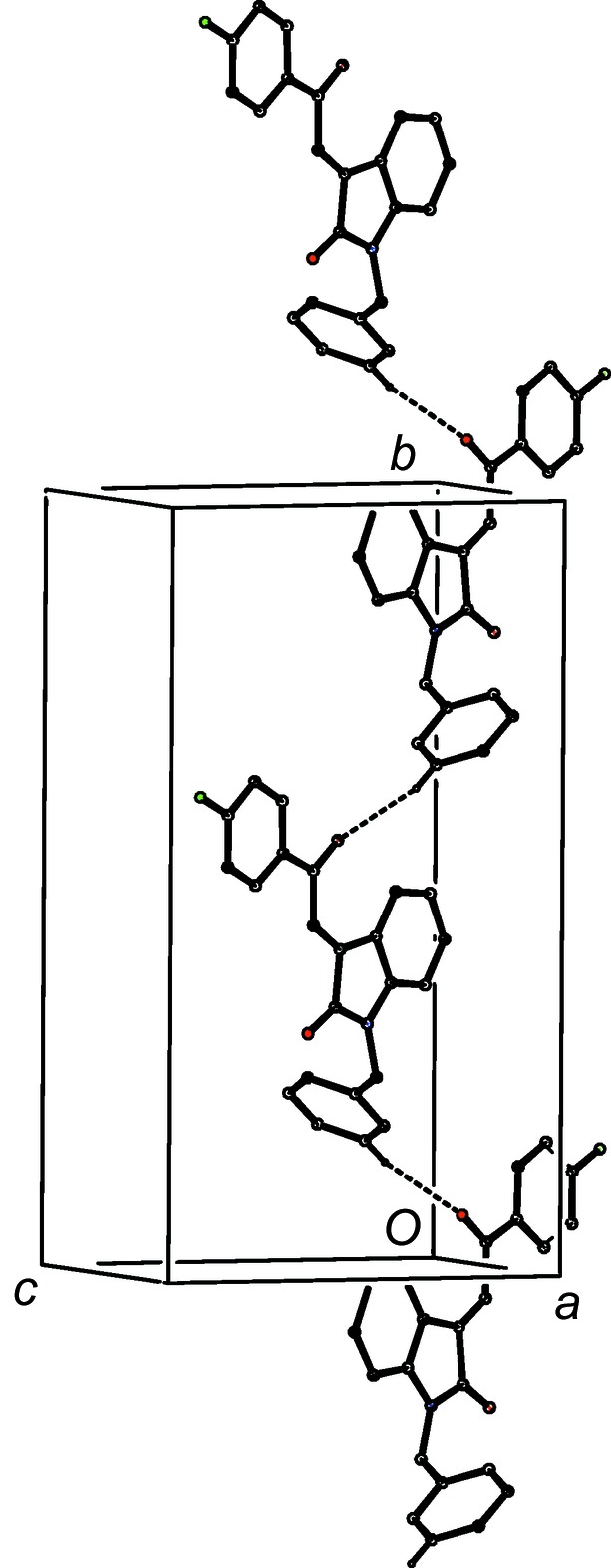
Part of the crystal structure of com­pound (II*a*), showing the formation of a hydrogen-bonded chain running parallel to [010]. Hydrogen bonds are drawn as dashed lines and, for the sake of clarity, H atoms not involved in the motif shown have been omitted.

**Figure 19:**
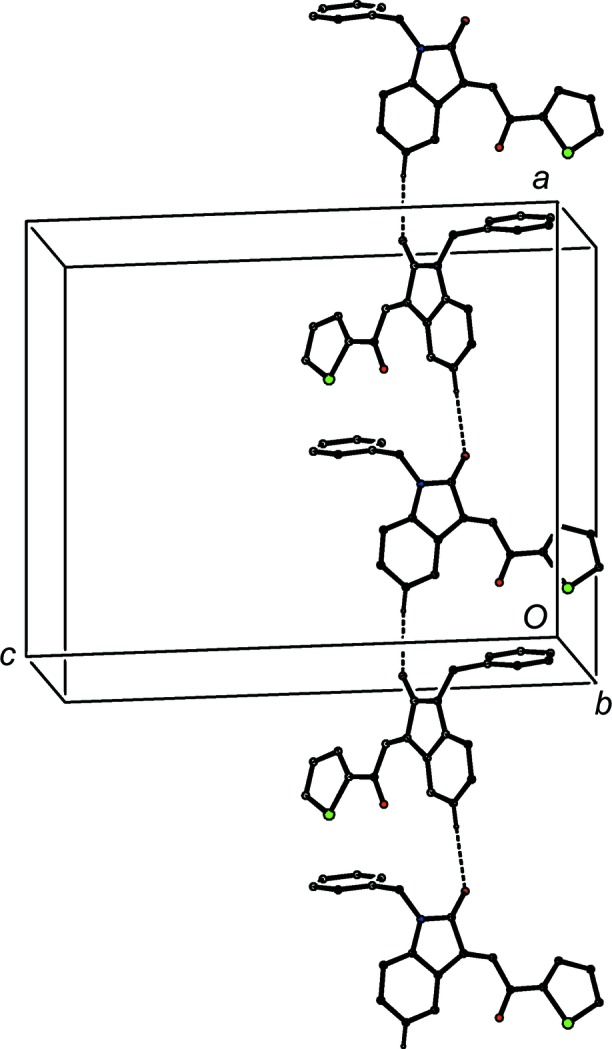
Part of the crystal structure of com­pound (II*g*), showing the formation of a hydrogen-bonded chain running parallel to [100]. Hydrogen bonds are drawn as dashed lines and, for the sake of clarity, the minor-disorder com­ponent and H atoms not involved in the motif shown have been omitted.

The absence of hy­droxy groups in the com­pounds of type (II) means that the hydrogen bonding in these structures is simpler than that found in com­pounds of type (I). Thus, for each of com­pounds (II*c*) and (II*h*), there are no significant inter­molecular hydrogen bonds, while in com­pound (II*a*), a single C—H⋯O hydrogen bond (Table 2 ▸) links mol­ecules which are related by a 2_1_ screw axis into a *C*(11) chain running parallel to the [010] direction (Fig. 18 ▸). The hydrogen bonding in com­pound (II*g*) is likewise very simple, with a single C—H⋯O hydrogen bond linking mol­ecules which are related by the *a*-glide plane at *z* = 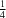 to form a *C*(7) chain running parallel to the [100] direction (Fig. 19 ▸).

**Figure 20:**
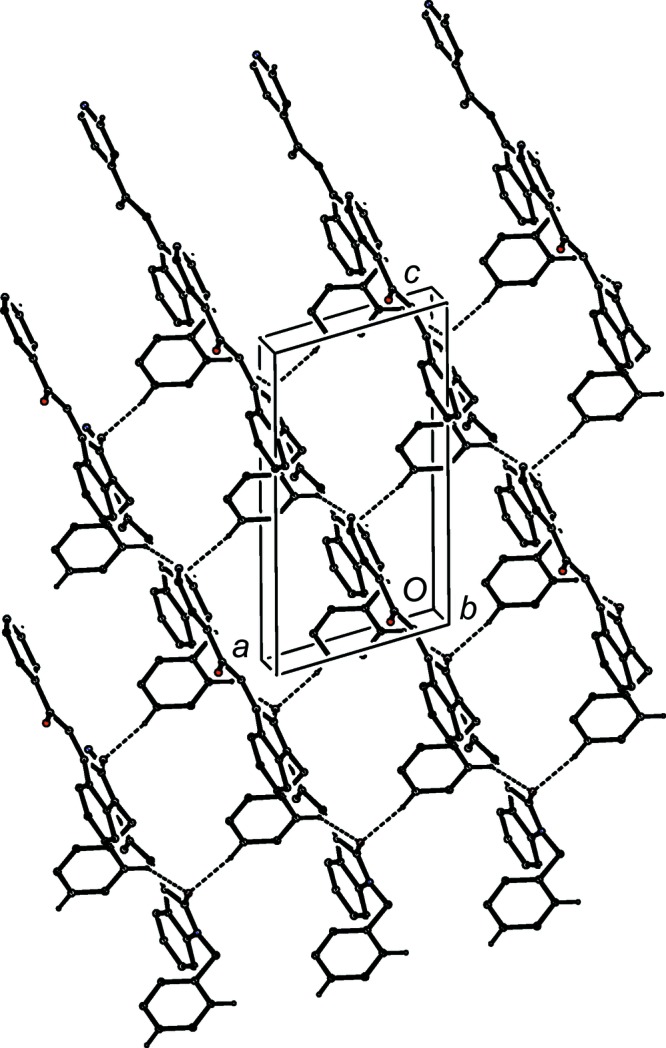
Part of the crystal structure of com­pound (II*e*), showing the formation of a sheet built from three C—H⋯O hydrogen bonds and lying parallel to (010). Hydrogen bonds are drawn as dashed lines and, for the sake of clarity, H atoms not involved in the motifs shown have been omitted.

A combination of three independent C—H⋯O hydrogen bonds links the mol­ecules of com­pound (II*e*) into a sheet lying parallel to (010) and lying in the domain 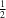 < *y* < 1.0 (Fig. 20 ▸); a second sheet, related to the first by inversion, lies in the domain 0 < *y* < 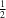, and adjacent sheets are linked by a C—H⋯π(arene) hydrogen bond, so generating a single three-dimensional framework structure.

**Figure 21:**
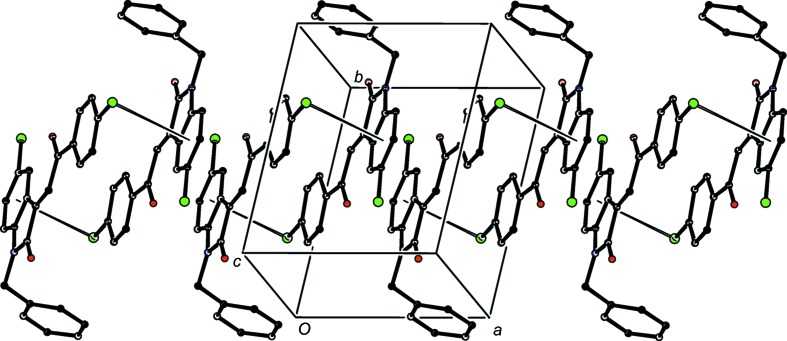
Part of the crystal structure of com­pound (II*h*), showing the formation of a chain of π-stacked dimers running parallel to the [100] direction. The Cl⋯(ring centroid) contacts are shown as tapered lines and, for the sake of clarity, H atoms have all been omitted.

Although there are no hydrogen bonds in the structure of com­pound (II*h*), the mol­ecules are nonetheless linked into a chain by the combination of a C—Cl⋯π(arene) inter­action and a π–π stacking inter­action. In the first of these inter­actions, atom Cl34 in the mol­ecule at (*x*, *y*, *z*) forms a short contact with the C3*A*/C4–C7/C7*A* ring in the mol­ecule at (−*x*, −*y* + 1, −*z* + 1), with geometric parameters Cl⋯*Cg* = 3.6055 (8) Å and C—Cl⋯*Cg* = 88.71 (5)°, where *Cg* represents the centroid of the aryl ring. The Cl⋯*Cg* distance here may be com­pared with the average value of 2.6° deduced from a database analysis of such contacts (Imai *et al.*, 2008 ▸), and this inter­action generates a cyclic centrosymmetric dimer. In addition, the C3*A*/C4–C7/C7*A* ring at (*x*, *y*, *z*) and the C321–C326 ring at (−*x* + 1, −*y* + 1, −*z* + 1) make a dihedral angle of only 7.58 (7)°. The ring-centroid separation is 3.7374 (9) Å and the shortest perpendicular distance from the centroid of one ring to the plane of the other is 3.3592 (6) Å, corresponding to a ring-centroid offset of *ca* 1.64 Å. The combination of these two inter­actions thus generates a chain of π-stacked dimers lying parallel to the [100] direction (Fig. 21 ▸).

The synthetic methodology described here is notable for its operational simplicity, broad substrate scope, good functional group com­patibility, and eco-com­patibility in terms of energy and waste. We note, in addition, that in each of the hy­droxy com­pounds (I*c*), (I*d*) and (I*f*), paired O—H⋯O hydrogen bonds generate 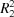
(10) motifs, which are centrosymmetric in each of (I*c*) and (I*d*), although the ring in (I*f*) exhibits no crystallographic symmetry. By contrast, the structure of pyri­dyl derivative (I*e*) contains no O—H⋯O hydrogen bonds (Table 2 ▸). It is inter­esting in this context to note that in a series of seven 3-alkyl-3-hy­droxy­indolin-2-ones, having no substituent on the N atom of the indoline ring, every structure contains a centrosymmetric 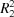
(10) ring embedded within a more com­plex supra­molecular assembly involving N—H⋯O hydrogen bonds and, in some cases, C—H⋯O and C—H⋯π(arene) hydrogen bonds also (Becerra *et al.*, 2010 ▸). Rings of the 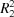
(10) type also occur in a number of related examples in the Cambridge Structural Database (CSD; Groom *et al.*, 2016 ▸), including examples having CSD refcodes MUMMAY (Chen *et al.*, 2009 ▸), TAWFAZ (Luppi *et al.*, 2005 ▸), TEQVUH (Luppi *et al.*, 2006 ▸) and YIFZIX (Xing *et al.*, 2007 ▸). Finally, we note that the reaction of isatin with cyclo­hexa­none involves both of the α-methyl­ene units of the cyclo­hexa­none, leading to the formation of 3,3′-[(1*RS*,3*SR*)-2-oxo­cyclo­hex­ane-1,3-di­yl]bis­[(3*RS*,3′*SR*)-3-hy­droxy­indolin-2-one] which was crystallized as a dehydrate (Becerra *et al.*, 2013 ▸). The organic com­ponents, which exhibit approximate, but noncrystallographic, mirror symmetry are linked by a combination of N—H⋯O and O—H⋯O hydrogen bonds to form sheets containing rings of 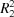
(8), 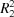
(16) and 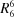
(40) types; these sheets are further linked by water mol­ecules, which themselves form cyclic centrosymmetric 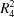
(8) tetra­mers.

## Supplementary Material

Crystal structure: contains datablock(s) global, Ic, Id, Ie, If, IIa, IIc, IIe, IIg, IIh. DOI: 10.1107/S2053229620004143/sk3748sup1.cif

Supporting information file. DOI: 10.1107/S2053229620004143/sk3748Icsup11.cml

Structure factors: contains datablock(s) Ic. DOI: 10.1107/S2053229620004143/sk3748Icsup2.hkl

Supporting information file. DOI: 10.1107/S2053229620004143/sk3748Idsup12.cml

Structure factors: contains datablock(s) Id. DOI: 10.1107/S2053229620004143/sk3748Idsup3.hkl

Supporting information file. DOI: 10.1107/S2053229620004143/sk3748Iesup13.cml

Structure factors: contains datablock(s) Ie. DOI: 10.1107/S2053229620004143/sk3748Iesup4.hkl

Supporting information file. DOI: 10.1107/S2053229620004143/sk3748Ifsup14.cml

Structure factors: contains datablock(s) If. DOI: 10.1107/S2053229620004143/sk3748Ifsup5.hkl

Supporting information file. DOI: 10.1107/S2053229620004143/sk3748IIasup15.cml

Structure factors: contains datablock(s) IIa. DOI: 10.1107/S2053229620004143/sk3748IIasup6.hkl

Supporting information file. DOI: 10.1107/S2053229620004143/sk3748IIcsup16.cml

Structure factors: contains datablock(s) IIc. DOI: 10.1107/S2053229620004143/sk3748IIcsup7.hkl

Supporting information file. DOI: 10.1107/S2053229620004143/sk3748IIesup17.cml

Structure factors: contains datablock(s) IIe. DOI: 10.1107/S2053229620004143/sk3748IIesup8.hkl

Supporting information file. DOI: 10.1107/S2053229620004143/sk3748IIgsup18.cml

Structure factors: contains datablock(s) IIg. DOI: 10.1107/S2053229620004143/sk3748IIgsup9.hkl

Structure factors: contains datablock(s) IIh. DOI: 10.1107/S2053229620004143/sk3748IIhsup10.hkl

Supporting information file. DOI: 10.1107/S2053229620004143/sk3748IIhsup19.cml

Spectroscopic data for compounds (Id)-(If), (Ih), (Ii), (IIa)-(IIc) and (IIe)-(IIi). DOI: 10.1107/S2053229620004143/sk3748sup20.txt

CCDC references: 1992762, 1992761, 1992760, 1992759, 1992758, 1992757, 1992756, 1992755, 1992754

## Figures and Tables

**Table:** Experiments were carried out at 100 K with Mo *K*α radiation using a Bruker D8 Venture diffractometer. Absorption was corrected for by multi-scan methods (*SADABS*; Bruker, 2016 ▸), except for (II*a*) and (II*e*), where *TWINABS* (Bruker, 2012) was used.

	(I*c*)	(I*d*)	(I*e*)
Crystal data			
Chemical formula	C_24_H_21_NO_4_	C_25_H_24_N_2_O_3_	C_22_H_18_N_2_O_3_
*M*_r_	387.42	400.46	358.38
Crystal system, space group	Monoclinic, *C*2/*c*	Triclinic, *P* 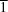	Triclinic, *P* 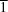
*a*, *b*, *c* (Å)	18.7572 (14), 13.2095 (10), 16.750 (2)	9.1028 (8), 10.6434 (9), 11.2539 (10)	7.8838 (5), 10.1766 (8), 11.8719 (9)
α, β, γ (°)	90, 105.374 (5), 90	88.988 (3), 68.422 (3), 81.352 (3)	87.554 (3), 75.996 (2), 69.428 (2)
*V* (Å^3^)	4001.7 (6)	1001.55 (15)	864.30 (11)
*Z*	8	2	2
μ (mm^−1^)	0.09	0.09	0.09
Crystal size (mm)	0.14 × 0.13 × 0.10	0.23 × 0.19 × 0.16	0.25 × 0.16 × 0.06

Data collection			
*T*_min_, *T*_max_	0.908, 0.991	0.946, 0.986	0.949, 0.994
No. of measured, independent and observed [*I* > 2σ(*I*)] reflections	18506, 4768, 3469	41110, 4590, 4033	55488, 4333, 3760
*R*_int_	0.051	0.037	0.045
(sin θ/λ)_max_ (Å^−1^)	0.659	0.650	0.670

Refinement			
*R*[*F* ^2^ > 2σ(*F* ^2^)], *wR*(*F* ^2^), *S*	0.047, 0.110, 1.02	0.038, 0.098, 1.05	0.038, 0.101, 1.07
No. of reflections	4768	4590	4333
No. of parameters	266	276	247
No. of restraints	0	0	0
H-atom treatment	H atoms treated by a mixture of independent and constrained refinement	H atoms treated by a mixture of independent and constrained refinement	H atoms treated by a mixture of independent and constrained refinement
Δρ_max_, Δρ_min_ (e Å^−3^)	0.30, −0.26	0.30, −0.22	0.41, −0.22

**Table:** 

	(I*f*)	(II*a*)	(II*c*)
Crystal data			
Chemical formula	C_24_H_19_NO_5_	C_23_H_16_FNO_2_	C_24_H_19_NO_3_
*M*_r_	401.40	357.37	369.40
Crystal system, space group	Triclinic, *P* 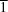	Monoclinic, *P*2_1_/*c*	Monoclinic, *P*2_1_/*n*
*a*, *b*, *c* (Å)	11.8136 (7), 12.4987 (10), 13.5976 (11)	7.6021 (6), 20.4880 (13), 10.9319 (7)	4.9743 (2), 29.1957 (13), 12.4406 (6)
α, β, γ (°)	93.084 (3), 101.883 (2), 95.055 (2)	90, 96.986 (3), 90	90, 100.914 (2), 90
*V* (Å^3^)	1951.7 (3)	1690.0 (2)	1774.05 (14)
*Z*	4	4	4
μ (mm^−1^)	0.10	0.10	0.09
Crystal size (mm)	0.25 × 0.16 × 0.06	0.14 × 0.14 × 0.10	0.45 × 0.06 × 0.04

Data collection			
*T*_min_, *T*_max_	0.957, 0.994	0.917, 0.990	0.948, 0.996
No. of measured, independent and observed [*I* > 2σ(*I*)] reflections	125792, 9691, 8134	3896, 3896, 2995	54500, 4142, 3643
*R*_int_	0.049	N/A	0.048
(sin θ/λ)_max_ (Å^−1^)	0.667	0.652	0.653

Refinement			
*R*[*F* ^2^ > 2σ(*F* ^2^)], *wR*(*F* ^2^), *S*	0.039, 0.106, 1.08	0.058, 0.134, 1.05	0.037, 0.095, 1.06
No. of reflections	9691	3896	4142
No. of parameters	547	245	254
No. of restraints	0	0	0
H-atom treatment	H atoms treated by a mixture of independent and constrained refinement	H-atom parameters constrained	H-atom parameters constrained
Δρ_max_, Δρ_min_ (e Å^−3^)	0.38, −0.24	0.29, −0.28	0.27, −0.23

**Table:** 

	(II*e*)	(II*g*)	(II*h*)
Crystal data			
Chemical formula	C_22_H_16_N_2_O_2_	C_21_H_15_NO_2_S	C_23_H_15_Cl_2_NO_2_
*M*_r_	340.37	345.40	408.26
Crystal system, space group	Monoclinic, *P*2_1_/*n*	Orthorhombic, *P* *b* *c* *a*	Triclinic, *P* 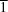
*a*, *b*, *c* (Å)	7.3457 (6), 18.0675 (16), 13.1813 (13)	17.5058 (14), 8.8163 (6), 21.2092 (16)	8.2010 (6), 9.7629 (7), 12.1740 (9)
α, β, γ (°)	90, 105.994 (3), 90	90, 90, 90	76.755 (3), 87.675 (3), 76.211 (3)
*V* (Å^3^)	1681.7 (3)	3273.4 (4)	921.34 (12)
*Z*	4	8	2
μ (mm^−1^)	0.09	0.21	0.37
Crystal size (mm)	0.16 × 0.15 × 0.12	0.15 × 0.07 × 0.05	0.40 × 0.16 × 0.07

Data collection			
*T*_min_, *T*_max_	0.878, 0.990	0.942, 0.989	0.924, 0.974
No. of measured, independent and observed [*I* > 2σ(*I*)] reflections	4167, 4167, 3281	32005, 4154, 3280	51788, 4558, 3867
*R*_int_	N/A	0.065	0.053
(sin θ/λ)_max_ (Å^−1^)	0.668	0.672	0.667

Refinement			
*R*[*F* ^2^ > 2σ(*F* ^2^)], *wR*(*F* ^2^), *S*	0.044, 0.121, 1.09	0.038, 0.092, 1.04	0.032, 0.082, 1.11
No. of reflections	4167	4154	4558
No. of parameters	237	239	253
No. of restraints	0	10	0
H-atom treatment	H-atom parameters constrained	H-atom parameters constrained	H-atom parameters constrained
Δρ_max_, Δρ_min_ (e Å^−3^)	0.29, −0.19	0.29, −0.31	0.44, −0.34

Computer programs: *APEX3* (Bruker, 2018 ▸), *SAINT* (Bruker, 2017 ▸), *SHELXT2014* (Sheldrick, 2015*a* ▸), *SHELXL2014* (Sheldrick, 2015*b* ▸) and *PLATON* (Spek, 2020 ▸).

**Table 2:** Hydrogen bonds and related short intra­molecular contacts (Å, °) for com­pounds (I*c*)–(I*f*), (II*a*), (II*e*) and (II*g*) *Cg*1, *Cg*2 and *Cg*3 represent the centroids of the C3*A*/C4–C7/C7*A*, C11–C16 and C13*A*/C14–C17/C17*A* rings, respectively.

	*D*—H⋯*A*		*D*—H	H⋯*A*	*D*⋯*A*	*D*—H⋯*A*
(I*c*)	O3—H3⋯O2^i^		0.86 (2)	2.10 (2)	2.9487 (15)	171.1 (18)
	C6—H6⋯O324^ii^		0.95	2.41	3.297 (2)	155
	C31—H31*B*⋯O2^i^		0.99	2.48	3.312 (2)	141
	C4—H4⋯*Cg*2^iii^		0.95	2.93	3.6101 (18)	130
	C14—H14⋯*Cg*1^iv^		0.95	2.82	3.709 (2)	156
(I*d*)	O3—H3⋯O2^v^		0.868 (18)	1.918 (18)	2.7630 (12)	164.2 (18)
	C7—H7⋯O32^vi^		0.95	2.44	3.3343 (16)	157
	C1—H1*B*⋯*Cg*1^vi^		0.99	2.96	3.8375 (14)	149
(I*e*)	O3—H3⋯N321^vii^		0.897 (17)	1.897 (17)	2.7915 (14)	174.9 (15)
	C4—H4⋯O3^v^		0.95	2.46	3.3842 (14)	164
	C7—H7⋯O32^viii^		0.95	2.51	3.3719 (14)	150
	C325—H325⋯O2^ix^		0.95	2.32	3.2578 (15)	171
	C322—H322⋯*Cg*1^ii^		0.95	2.68	3.5294 (14)	149
(I*f*)	O13—H13⋯O22		0.874 (17)	1.912 (17)	2.7794 (12)	171.1 (17)
	O23—H23⋯O12		0.874 (17)	1.912 (17)	2.7794 (12)	171.1 (17)
	C131–H13*A*⋯O22^vi^		0.99	2.35	3.3075 (16)	161
	C147—H147⋯O141^x^		0.95	2.56	3.4776 (18)	163
	C231—H23*A*⋯O12^v^		0.99	2.37	3.3107 (15)	159
	C242—H24*A*⋯O23^ii^		0.99	2.53	3.4889 (19)	163
	C142—H14*A*⋯*Cg*3^ii^		0.99	2.53	3.3289 (15)	137
(II*a*)	C15—H15⋯O32^xi^		0.95	2.49	3.278 (3)	141
(II*e*)	C14—H14⋯O2^xii^		0.95	2.32	3.234 (3)	161
	C16—H16⋯O2^xiii^		0.95	2.45	3.230 (2)	140
	C326—H326⋯O2^xiv^		0.95	2.58	3.494 (2)	162
	C6—H6⋯*Cg*2^xiii^		0.95	2.64	3.566 (2)	165
(II*g*)	C5—H5⋯O2^xv^		0.95	2.59	3.5058 (19)	161
	C323—H323⋯*Cg*2^xvi^		0.95	2.93	3.744 (3)	145

Symmetry codes: (i) −*x* + 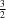, −*y* + 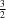, −*z* + 1; (ii) *x*, *y* − 1, *z*; (iii) *x*, −*y* + 1, *z* + 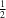; (iv) −*x* + 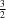, −*y* + 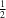, −*z* + 1; (v) −*x* + 1, −*y* + 1, −*z* + 1; (vi) −*x*, −*y* + 1, −*z* + 2; (vii) *x*, *y* + 1, *z*; (viii) −*x* + 1, −*y* + 1, −*z*; (ix) −*x*, −*y* + 1, −*z* + 1; (x) −*x*, −*y*, −*z* + 1; (xi) −*x* + 1, *y* − 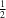, −*z* + 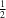; (xii) *x* + 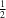, −*y* + 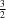, *z* − 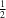; (xiii) *x* − 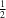, −*y* + 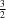, *z* − 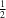; (xiv) *x* + 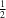, −*y* + 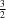, *z* + 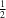; (xv) *x* − 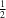, *y*, −*z* + 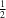; (xvi) −*x* + 1, *y* + 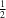, −*z* + 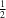.

**Table 3:** Selected torsion angles (°) for com­pounds (I*c*)–(I*f*)

Parameter	(I*c*)	(I*d*)	(I*e*)	(I*f*), mol­ecule 1	(I*f*), mol­ecule 2
				(*x* = 1)	(*x* = 2)
C*x*2—N*x*1—C*x*1—C*x*11	103.75 (16)	102.09 (12)	95.90 (12)	119.11 (13)	108.60 (13)
N*x*1—C*x*1—C*x*11—C*x*12	−28.5 (2)	−40.32 (15)	−76.98 (13)	−54.19 (16)	−27.57 (17)
N*x*1—C*x*2—C*x*3—C*x*31	−126.14 (13)	−133.07 (10)	−124.96 (9)	−123.72 (10)	−126.04 (10)
C*x*2—C*x*3—C*x*31—C*x*32	52.57 (17)	58.79 (13)	61.83 (12)	50.92 (13)	51.93 (13)
C3—C31—C32—C321	−176.52 (13)	179.12 (10)			
C3—C31—C32—C324			−179.45 (10)		
C31—C32—C321—C322	175.64 (14)	−176.22 (10)			
C31—C32—C324—C323			−149.73 (12)		
C*x*3—C*x*31—C*x*32—C*x*45				−174.30 (10)	−174.80 (10)
C*x*31—C*x*32—C*x*45—C*x*44				176.61 (10)	178.65 (11)

**Table 4:** Selected torsion angles (°) for com­pounds (II*a*), (II*c*), (II*e*), (II*g*) and (II*h*)

Parameter	(II*a*)	(II*c*)	(II*e*)	(II*g*)	(II*h*)
C2—N1—C1—C11	111.4 (2)	102.92 (13)	90.35 (19)	94.94 (16)	98.83 (15)
N1—C1—C11—C12	−41.8 (3)	−61.57 (14)	−1.1 (2)	−65.13 (18)	−62.18 (16)
N1—C2—C3—C31	−176.39 (19)	−177.72 (10)	179.61 (15)	177.30 (12)	−179.37 (12)
C2—C3—C31—C32	176.1 (2)	178.62 (11)	−178.71 (16)	178.91 (13)	177.52 (13)
C3—C31—C32—C321	−176.0 (2)	172.77 (11)			177.57 (14)
C3—C31—C32—C322				−175.99 (14)	
C3—C31—C32—C324			179.55 (16)		
C31—C32—C321—C322	−178.2 (2)	169.40 (10)			175.80 (12)
C31—C32—C322—S321				167.55 (10)	
C31—C32—C324—C323			173.76 (15)		
